# The step-up protocol increases clinical pregnancy rates compared with the step-down in patients with unexplained infertility. A randomized controlled trial

**DOI:** 10.5935/1518-0557.20210112

**Published:** 2022

**Authors:** Ana Robles, Sonia Gayete-Lafuente, Maria Prat, Mireia Gonzalez-Comadran, Miguel Ángel Checa

**Affiliations:** 1 Reproductive Endocrinology Unit. Department of Obstetrics and Gynecology. Hospital del Mar de Barcelona. Universitat Autònoma de Barcelona (UAB), Barcelona, Spain

**Keywords:** ovarian stimulation, step-up protocol, step-down protocol, intrauterine insemination, unexplained infertility

## Abstract

**Objective:**

Unexplained infertility is a relevant indication for controlled ovarian stimulation associated to intrauterine insemination. The "step-up" and "step-down" gonadotropin-based protocols were designed to reduce multiple pregnancy and ovarian hyperstimulation syndrome in polycystic ovary syndrome patients, but there is no related evidence in normoovulatory women undergoing intrauterine insemination. Our aim was to compare the efficacy and safety of both protocols with intrauterine insemination in unexplained infertility patients.

**Methods:**

Randomized clinical trial including 145 women with unexplained infertility randomly following the step-up (n=73) or step-down (n=72) protocol. In the step-up group, patients started on day 3 of a spontaneous cycle administrating recombinant FSH 75IU sc/day, increasing it to 150IU if no response after 7 days. In the step-down, patients started administrating 150IU sc/day, constantly decreasing it to 75IU after 5 days. Recombinant hCG was administered when a follicle reached ≥18mm diameter.

**Results:**

Clinical pregnancy rate was higher in the step-up group than in the step-down (20.5% *vs* . 8.3%; *p* =0.037). Significant differences between step-up and step-down protocols were found regarding days of rFSH administration (8.83±4.01% *vs* . 7.42±2.18%; *p* =0.001) and cancellation rate due to hyper response (8.21% *vs* . 25%; *p* =0.05). No differences were detected in miscarriage rates, multiple pregnancy rates/cycle and hyper stimulation syndrome incidence.

**Conclusions:**

The step-up protocol is longer-lasting but more effective obtaining pregnancies than the step-down in patients with unexplained infertility undergoing intrauterine insemination. This effect could be explained by lower cancellation rates due to ovarian hyper response than the step-down protocol, with no differences in ovarian hyper stimulation syndrome incidence.

## INTRODUCTION

Unexplained infertility is defined as the lack of a cause for conceiving failure after standard infertility testing, and affects 30-50% of couples unable to conceive (Ray *et al* ., 2012). It includes idiopathic cases, usually treated by ovarian stimulation with gonadotropins associated with intrauterine insemination (IUI) in order to increase pregnancy likelihood by rising the number of eggs available for fertilization up to 2-3 (Steures *et al* ., 2006). Nevertheless, these strategies increase the multifollicular development risk, leading to higher incidence of multiple pregnancy and ovarian hyper stimulation syndrome (OHSS) (Sagle *et al* ., 1991; Homburg *et al* ., 1995; Sociedad Española de Fertilidad, 2017).

The precise mechanisms of mono/oligofollicular development goal for IUI are still unknown, making it difficult to design safe pharmacological strategies. In this context, two different controlled ovarian stimulation protocols, known as "step-up" and "step-down", were designed to avoid multifollicular growth. Both protocols are based on adapted sequential gonadotropin dosage changing to induce a more physiological ovulation, either by increasing doses in the step-up protocol or by decreasing them in the step-down. These regimens have been widely but heterogeneously evaluated in polycystic ovary syndrome (PCOS) patients, with major risk of multifollicular growth because of their elevated ovarian reserve and particularly extreme sensitivity to exogenous gonadotropins (Sagle *et al* ., 1991; Homburg *et al* ., 1995; Kamrava *et al* ., 1982; Seibel *et al* ., 1984; Polson *et al* ., 1987; Buvat *et al* ., 1989; Shoham *et al* ., 1991; Dale *et al* ., 1993; van Santbrink *et al* ., 1995; White *et al* ., 1996). Although both protocols have shown to be successful in reducing the number of growing follicles versus other strategies, the authors found conflicting results when comparing their efficacy on time to follicular recruitment, hormone concentrations, and resulting pregnancy rates (Mizunuma *et al* ., 1991; van Santbrink & Fauser, 1997; Andoh *et al* ., 1998; Balasch *et al* ., 2001; Christin-Maitre & Hugues, 2003). Moreover, their potential benefits have not been evaluated in non-PCOS patients, who represent the leading cause of IUI performance.

As decreasing multifollicular growth may reduce the risk of multiple pregnancy and OHSS in all patients, testing the step-up and step-down protocols in non-PCOS may contribute to diminish the adverse events related to IUI. In this study, our aim was to compare these protocols in patients with unexplained infertility undergoing IUI.

## MATERIALS AND METHODS

### Study design

This study is a prospective randomized parallel controlled clinical trial with 1:1 allocation performed at Hospital del Mar, Barcelona, and designed to compare the efficacy and safety of two different gonadotropin-based ovarian stimulation protocols in unexplained infertile couples undergoing IUI. It was approved by the local Clinical Research Ethics Committee, and all patients signed informed consent to take part in. No changes to methods were conducted after the trial commencement.

ClinicalTrials.gov Identifier: NCT01376999.

### Participants

Eligible individuals were women aged 18-40 years old who had been diagnosed of unexplained infertility and planning IUI, selected for enrolment between June 2011 and February 2013 after an extended medical history record and baseline evaluation. In the first visit, patients were explained the purpose, requisites and procedures of the study, and no invasive tests were indicated before obtaining informed consents.

Unexplained infertility was defined as constant attempt to become pregnant for 1 year before the initial fertility evaluation, with no clear cause of conceiving failure after routine infertility study procedures in our unit. In all patients, tubal permeability was demonstrated by hysterosalpingography. Ovarian function was evaluated using a basal serum determination of FSH, LH, estradiol (E2) and prolactin; only patients with basal FSH <10mUI/ml and normal prolactin levels and were included. Moreover, ovarian reserve was evaluated with an antimullerian hormone (AMH) determination, as well as seric inhibin B levels and ultrasonographic antral follicles count (AFC). Women with oligoamenorrhea or meeting the criteria for PCOS diagnose based on Rotterdam Criteria 2004 were excluded (Rotterdam ESHRE/ASRM-Sponsored PCOS Consensus Workshop Group, 2004). Uterine integrity was evaluated by vaginal ultrasound. Cases of benign uterine pathologies and dysfunctional bleeding were excluded, as well as history of >3 previous cycles of IUI. Male factor was studied with at least one seminogram evaluated according to WHO criteria 2010; only patients with partners presenting >5 millions of motile sperm were included. Other exclusion criteria were <20/>30 body mass index (BMI), any chronic systemic disease, and any positive serology to HIV, HVC/HVB or syphilis.

### Interventions

Step-up protocol group: Women received rFSH (Gonal pen^(r))^ 75IU sc daily, starting on day 3 of a spontaneous cycle. Vaginal ultrasound was performed on day 7 of treatment, and the rFSH dose was increased to 150 IU if no response was observed. 

Step-down protocol group: Women started with rFSH (Gonal pen^(r))^ 150IU sc daily from day 3 of a spontaneous cycle. Vaginal ultrasound was performed on day 5 of treatment, and the rFSH dose was decreased to 75 IU in all cases. 

In both protocols, from the respective first control visit, vaginal ultrasound was performed every 48 hours until ≥1 follicle reached ≥18 mm diameter, so a single dose of rhCG (Ovitrelle pen^®^) 250 µg sc was given. Cycles were cancelled if ≥4 follicles of ≥14mm were observed. IUI was performed 36 hours after rhCG administration, previous semen capacitating. In both groups luteal phase was supported with progesterone (Utrogestan^®^) 200mg/24 hours after IUI.

**Figure 1:**
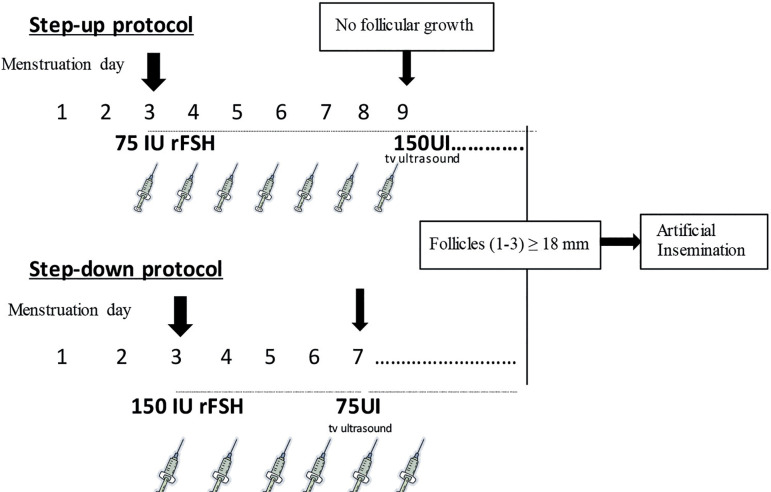
Step-up and Step-down ovarian stimulation protocols

Figure 1 summarizes the study protocols.

### Outcomes

The primary outcomes were clinical pregnancy rates; defined according to the ART terminology (Zegers-Hochschild *et al* ., 2009). Also multiple pregnancy rates, OHSS rates and miscarriage rates were registered.

Secondary outcomes were: duration of the completed ovarian stimulation for each protocol (days), total amount of rFSH used (IU), number of growing follicles, diameter of growing follicles (mm), serum E2 levels achieved at day of rhCG (pg/ml), and hyper response rate leading to IUI cancellation in each group of patients.

### Sample size

Predefining an alfa and beta risk of 0.05 and 0.20, respectively, in a bilateral contrast, we calculated a sample size of 75 patients in each group to find statistically significant differences >20% in pregnancy rates between groups. A total 10% follow-up loss was accepted.

### Randomization

Randomization was generated by computerization on a web-based program, then automatically exported to an excel program.

### Statistical analysis of results

An unpaired Student´s t-test of variance was performed when appropriate. For primary outcomes, we applied an intention-to-treat approach to get clinically relevant findings for medical practice. Data were analyzed by SPSS software (18.0 version, Chicago, USA) assuming a statistically significant level of 5% (*p* <0.05).

### Cost analysis

The financial costs of both stimulation protocols and the costs per pregnancy obtained in each group of patients were calculated as follows: 

-Cost of a stimulation protocol = (Cost of gonadotropins x mean of the total sFSH used units) + €50 Ovitrelle as trigger medication + €300 cost of seminal preparation and in-office insemination including cannula, speculum, etc.

-Cost per pregnancy obtained = (Cost of the protocol /patient x nº of stimulated patients) / pregnancies achieved.

## RESULTS

**Figure 2:**
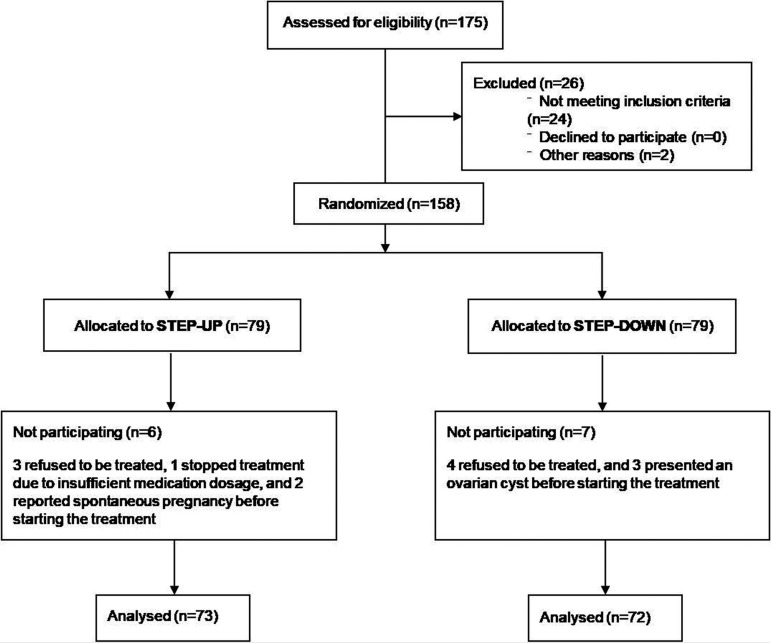
Participant flow chart

A total of 158 eligible patients with unexplained infertility were recruited. 79 were randomly allocated in the step-up group and 79 in the step-down. Finally, 73 cycles in the step-up group and 72 in the step-down were initiated and analyzed. Flow chart is shown in Figure 2.

Basal characteristics of patients were comparable between groups, as reported in Table 1.

**Table 1:** Basal characteristics of 145 patients with unexplained infertility. Women were randomized for step-up or step-down protocol.

	Step-down (n=72) (Mean ± SD*)	Step-up (n=73) (Mean ± SD)
Age (years)	34.69±3.87	34.73±3.81
FSH (mIU/ml)	6.79±1.57	7.00±1.75
LH (mIU/ml)	6.20±2.21	6.63±2.15
Estradiol (pg/ml)	48.72±23.65	53.77±36.18
AMH (mcg/L)	3.32±3.20	2.54±2.59
Inhibin B (pg/ml)	87.78±101.16	68.61±49.58
Right antral follicles	5.68±1.99	6.42±2.65
Left antral follicles	6.32±3.09	6.48±3.21

* SD=standard deviation.

We observed a significantly higher clinical pregnancy rate in the step-up group than in the step-down (20.5% *vs* . 8.3%; *p* =0.037). Twin pregnancies showed a non-significant tendency to be higher in the step-down group than in step-up (16.7% *vs* . 6.7%; *p* =0.5). The miscarriage rate was similar between both regimens (13.3% *vs* . 16.7%; *p* =1.0).

The number of intermediate grown follicles (14-17mm diameter at the time of hCG) did significantly differ between the step-up and the step-down protocols (1.48±2.26 *vs* . 2.32±2.40; *p* =0.019). Moreover the cases of ovarian hyper response were significantly higher with the step-down protocol than with the step-up (18 vs. 6), leading to a major cancellation rate (25% *vs* . 8.21%; *p* =0.005). When comparing the patients undergoing IUI only, without considering the cancelled cycles, we found no statistical differences regarding clinical pregnancy rates between the step-up and the step-down group (22.4% *vs* . 11.1%; *p* =0.103).

No cases of severe OHSS were documented, although 1 patient suffered a mild case in the step-up group and 3 patients in the step-down (1.23% *vs* . 4.16%; *p* =0.363). Those four cases had a favorable evolution and did not require medical intervention. All outcomes are presented in Table 2.

**Table 2:** Clinical results among de two study groups.

	Step-up (n=73) N (positives/total, %)	Step-down (n=72) N (positives/total, %)	*p*-value
Clinical pregnancy	15 (20.5)	6 (8.3)	0.037
Twin pregnancy	1 (6.7)	1 (16.7)	0.5
Miscarriages	2 (13.3)	1 (16.7)	1.0
Treatment duration (days)	8.83±4.01	7.42±2.18	0.001
Total amount of rFSH (IU)	874.5±300.75	856.5±327	0.34
Intermediate follicles (14-17mm)	1.48±2.26	2.32±2.40	0.019
Estradiol at day of rhCG (pg/ml)	640.83±501.4	845.31±564.565	0.034
Hyper response. cancelled	7 (8.21)	18 (25)	0.005
OHSS	1 (1.37)	3 (4.17)	0.363

*N=total number.

The step-up protocol financial cost was €682.31 per patient [(€0.38/ IU Gonal-F x 874.5 IU) + €50 Ovitrelle + €300 insemination]; while the step-down protocol cost was €675.47 per patient [(€0.38/ IU Gonal-F x 856.5 IU) + €50 Ovitrelle + €30].

The financial cost per pregnancy obtained in the step-up group was €3.320.58 [(€682.31 x 73 patients) / 15 pregnancies achieved]; while the cost per pregnancy obtained in the step-down was €8,105.64 [(€675.47 x 72 patients) / 6 pregnancies achieved].

Both compared protocols represent similar prices per patient, but the cost per pregnancy in the Step-up group is 2.44 times lower. Therefore, we found that the Step-up protocol is not only associated to a higher clinical pregnancy rate in the study population (20.5% *vs* . 8.3%; *p* -value=0.037), but also remarkably cost-efficient. Given that the Step-down protocol relates with a significantly lower clinical pregnancy rate, higher cancellation rate and much higher financial cost per pregnancy, we reinforce our recommendation of using the step-up protocol in patients with unexplained infertility undergoing IUI.

## DISCUSSION

This study found that the step-up protocol is more effective achieving clinical pregnancy by IUI than the step-down in unexplained infertile couples, presenting a lower cancellation rate due to ovarian hyper response, and without differences in the assessed adverse events.

To date, there were no studies comparing the efficacy and safety of the step-up and the step-down protocols in this group of patients, but both regimens have been widely studied in PCOS patients, mainly regarding follicular growth outcomes (Mizunuma *et al* ., 1991; van Santbrink & Fauser, 1997; Andoh *et al* ., 1998; Balasch *et al* ., 2001; Christin-Maitre & Hugues, 2003; Hugues *et al* ., 1996). While some authors reported that the step-up protocol seems safer and resulted in more monofollicular cycles, others totally differ in their findings supporting the step-down strategy. There is also controversy about the treatment duration, rFSH needed and E2 levels achieved. The only authors also analyzing the related pregnancy rates, found no differences between the two protocols (Christin-Maitre & Hugues, 2003). However, the important heterogeneity in the design of these studies, including different kind and doses of administered gonadotropins, could induce to contradictory conclusions. Moreover, PCOS patients are at particular higher risk of multifollicular development, so of multiple pregnancy and OHSS, than normoovulatory patients as the included in our study (Sagle *et al* ., 1991; Homburg *et al* ., 1995).

Although many patient-specific factors as age, AFC, high AMH levels, BMI and previous response to ovulation induction can influence the number of growing follicles per cycle (Checa *et al* ., 2010; Jeon *et al* ., 2013; Thijssen *et al* ., 2017; Speyer *et al* ., 2013); these baseline characteristics were comparable between study groups in our trial.

The pregnancy rates we obtained in the step-up group were superior than the reported for IUI in our media according to the last Spanish national register (Sociedad Española de Fertilidad, 2017), around 15.6% in women aged <40 years old and 9.9% in those >40. Contrarily, pregnancy rates in the step-down group were lower than expected. The higher cancellation rate in the step-down group might have influenced the pregnancy rate difference reported, favoring the step-up protocol. This difference is not found when comparing the cycles finally undergoing IUI only, which sustains our rationale and represents a relevant finding for clinical practice. Differences were neither found in AMH levels and basal AFC between the patients with cancelled cycles due to hyper response and those who underwent IUI. In ovulatory women, starting the treatment with higher rFSH doses initially recruits a major number of follicles explaining the greater multifollicular response in the step-down group, which does not seem to decrease by diminishing rFSH later in the cycle (Tan *et al* ., 2005). Similarly, increasing rFSH dose during the step-up protocol course does not seem to rectify an initial monofollicular growth, thus not leading to hyper response (van Hooff *et al* ., 1993; Hock *et al* ., 1998; Khalaf *et al* ., 2002).

The higher multifollicular developments in the step-down group causing a 25% of cancellations are consistent with some reports from studies on PCOS patients (Andoh *et al* ., 1998), differing from others (van Santbrink & Fauser, 1997; Andoh *et al* ., 1998; Balasch *et al* ., 2001). Van Santbrink & Fauser (1997) observed a higher monofollicular development in PCOS clomiphene-resistant patients following the step-down protocol compared with the ones following the step-up (88% *vs* . 56%; *p* =0.04). This could be attributed to their basal anovulatory condition, better responding to higher initial doses followed by a more physiological decrease of rFSH than to a stepping up approach possibly leading them to a higher number of cancellations (Koundouros, 2008). However, our study included normoovulatory women only, with better chances of initial monofollicular response to rFSH lower doses than some PCOS (Sagle *et al* ., 1991; Homburg *et al* ., 1995). Also, our step-up regimen resulted in a longer-lasting induction with comparable rFSH units required versus the step-down, similarly to what other authors reported (van Santbrink & Fauser, 1997; Christin-Maitre & Hugues, 2003). 

We found no difference in multiple pregnancy rates between groups, although the sample size of this study was not specifically calculated to detect so. In contrast, a trial involving 1682 ovarian stimulated IUI cycles reported a multiple pregnancy rate of 10.5%, associating the rate of multiplets to the number of follicles >14mm diameter observed the day of hCG (3,6% if 1 follicle >14mm, 10% if 2, 17% if 3, and 45% if >3; *p* =0.0001) (Ghesquiere *et al* ., 2007). These data are in agreement with the recently reported by ESHRE (European Society of Human Reproduction and Embriology) from 169.952 IUI cycles analyzed, reporting 9.5% twins in women <40 years old (De Geyter *et al* ., 2018). We did not notify any case of severe OHSS, which was expected considering the limited number of growing follicles. Our OHSS incidence was similar to those previously reported (Golan *et al* ., 1989).

One limitation of our study was the impossibility to evaluate live born rates because an important follow-up loss of cases once clinical pregnancy was confirmed and patients transferred to local hospitals.

Finally, both compared protocols represent similar prices per patient in our setting, but the cost per pregnancy in the step-up group was 2,44 times lower. Therefore, we found that the step-up protocol was not only associated to a higher clinical pregnancy rate in the study population, but also remarkably cost-efficient. In contrast, the step-down protocol related with a higher cancellation rate and much higher financial cost per pregnancy; hence we reinforce our recommendation against it in the study population.

In conclusion, this study represents the first RCT comparing the step-up versus the step-down ovarian stimulation protocol in patients with unexplained infertility undergoing IUI. In this population, the step-up protocol obtains a better pregnancy rate with a significantly lower cancellation rate than the step-down, without differences in adverse outcomes as multiple pregnancy and OHSS. The step-up group also appears to be considerably cost-efficient. Therefore, at present time, the step-up protocol should be the first choice of gonadotropin therapy for IUI cycles in patients with unexplained infertility.
